# Reductions in Hydrogen Sulfide and Changes in Mitochondrial Quality Control Proteins Are Evident in the Early Phases of the Corneally Kindled Mouse Model of Epilepsy

**DOI:** 10.3390/ijms23031434

**Published:** 2022-01-27

**Authors:** Christi Cho, Maxwell Zeigler, Stephanie Mizuno, Richard S. Morrison, Rheem A. Totah, Melissa Barker-Haliski

**Affiliations:** 1 Department of Medicinal Chemistry, University of Washington, Seattle, WA 98195, USA; cwcho@uw.edu (C.C.); maxwell.zeigler@gmail.com (M.Z.); rtotah@uw.edu (R.A.T.); 2 Department of Pharmacy, University of Washington, Seattle, WA 98195, USA; stephaniemizuno@gmail.com; 3 Department of Neurology, University of Washington, Seattle, WA 98195, USA; yael@uw.edu

**Keywords:** epilepsy, hydrogen sulfide, corneally kindled mice, mitochondrial dysfunction, oxidative stress, LC-MS/MS, temporal lobe epilepsy, neurological disorder, gasotransmitter

## Abstract

Epilepsy is a heterogenous neurological disorder characterized by recurrent unprovoked seizures, mitochondrial stress, and neurodegeneration. Hydrogen sulfide (H_2_S) is a gasotransmitter that promotes mitochondrial function and biogenesis, elicits neuromodulation and neuroprotection, and may acutely suppress seizures. A major gap in knowledge remains in understanding the role of mitochondrial dysfunction and progressive changes in H_2_S levels following acute seizures or during epileptogenesis. We thus sought to quantify changes in H_2_S and its methylated metabolite (MeSH) via LC-MS/MS following acute maximal electroshock and 6 Hz 44 mA seizures in mice, as well as in the early phases of the corneally kindled mouse model of chronic seizures. Plasma H_2_S was acutely reduced after a maximal electroshock seizure. H_2_S or MeSH levels and expressions of related genes in whole brain homogenates from corneally kindled mice were not altered. However, plasma H_2_S levels were significantly lower during kindling, but not after established kindling. Moreover, we demonstrated a time-dependent increase in expression of mitochondrial membrane integrity-related proteins, OPA1, MFN2, Drp1, and Mff during kindling, which did not correlate with changes in gene expression. Taken together, short-term reductions in plasma H_2_S could be a novel biomarker for seizures. Future studies should further define the role of H_2_S and mitochondrial stress in epilepsy.

## 1. Introduction

Mitochondria are the predominant source of reactive oxygen species underlying oxidative neuronal damage, including damage observed in the brains of patients with acquired epilepsy [1]. Oxidative stress and mitochondrial dysfunction can directly induce apoptosis or necrosis; this aberrant neuronal loss can then increase seizure susceptibility [2]. One mechanism through which the oxidative damage can be mitigated is the scavenging of reactive oxygen and reactive nitrogen species by the endogenous gasotransmitter hydrogen sulfide (H_2_S). H_2_S is an important signaling molecule involved in various biological processes, including cytoprotection, anti-inflammation, oxygen sensing, neuromodulation, and neuroprotection [3,4,5,6]. The antioxidant function of H_2_S has been extensively studied. For example, H_2_S can mitigate oxidative damage through an increase in intracellular glutathione levels by modulating the activity of the cystine transporter system [7,8]. H_2_S can also alter the activity of key antioxidant enzymes, including glutathione peroxidase, superoxide dismutase, and catalase to attenuate oxidative stress [9]. Additionally, H_2_S is capable of indirectly upregulating the expression of several antiapoptotic transcription factors and mitochondrial biogenesis genes to promote cell survival [10]. In mammalian tissue, H_2_S is mainly synthesized via cystathionine β-synthase (CBS), cystathionine γ-lyase (CTH or CSE), and 3-mercaptopyruvate sulfurtransferase (3-MST or MPST) [10,11,12,13,14]. CBS and 3-MST are major sources of H_2_S production in the brain [15,16,17]. The substance 3-MST is localized in the cytosol and mitochondria, while CBS and CSE are localized in the cytosol but have been shown to translocate to the mitochondria under stress conditions [18]. Non-enzymatic production of H_2_S requiring a cysteine donor, iron, and vitamin B6 also contributes to endogenous H_2_S levels in red blood cells and potentially the brain [19]. 

Disruptions in mitochondrial membrane integrity dynamics and synaptic dysfunction are both associated with, and causally related to, epilepsy. While disturbances in energy metabolism and ATP production have received significant attention in the pathogenesis of epilepsy [20,21], abnormalities in mitochondrial shape, size, and distribution may also influence neurological disease [22]. The molecular regulators of mitochondrial membrane integrity dynamics, including fusion and fission (i.e., fragmentation) may possess important roles in neuronal survival [22,23]. Excessive fission is frequently reported in various neurological diseases, including in rodent epilepsy models [24]. The well-conserved dynamin-related GTPases, including dynamin-related protein 1 (Drp1), promote fission in conjunction with mitochondrial fission factor (Mff), one of at least two mitochondrial proteins involved in recruitment of Drp1 from the cytosol to the outer mitochondrial membrane. Endophilin B1 (Endo-B1) activates stress-response proteins (e.g., Bax), to promote cytochrome c release and apoptosis [25], and Endo-B1 has been shown to be neuroprotective in other neurological disease models [26,27]. The neuron-specific isoforms Endo-B1-B/C participates in the trafficking of the TrkA receptor, promoting receptor internalization and recycling in early endosomes and preventing endosome–lysosome fusion [28]. Furthermore, histone deacetylase 2 (HDAC2) regulates the expression of Endo-B1, particularly neuron-specific isoforms B/C [26,27], and is thus a non-mitochondrial marker of mitochondrial function, which may prevent neuronal pathology [29]. However, how these molecular regulators are dynamically altered during the development of chronic seizures or epilepsy in various animal models has yet to be established. Furthermore, both Drp1 and Mff are also involved in synaptic vesicle recycling, interacting with clathrin to promote endosome formation [30], implicating mitochondrial fission proteins in the regulation of synaptic plasticity, and network remodeling during the development of a hyperexcitable neuronal network, i.e., epilepsy. 

The function of H_2_S in preserving mitochondrial function and its effect on mitochondrial dynamics has been previously established. A 3-MST-mediated increase in H_2_S levels attenuates mitochondrial damage in cerebral endothelial cells during ischemia/reperfusion injury [17]. Exogenous H_2_S preserves mitochondrial function following myocardial ischemia reperfusion injury in mice by mitigating infarct size and promoting cell survival in a dose-dependent manner [31]. H_2_S promotes mitochondrial fusion and inhibits fission by modulating genes involved in mitochondrial membrane integrity dynamics, including Drp1 and MFN2 [32]. At higher concentrations, however, H_2_S also elicits cell toxicity by promoting apoptosis through the recruitment of Bax to the mitochondria. Furthermore, elevated H_2_S levels result in cerebellar mitochondrial swelling and a decrease in the mitochondrial membrane potential in rat models of ethylmalonic encephalopathy highlighting the biphasic nature of H_2_S-induced cellular protection and toxicity [10,33]. 

Despite clear understanding of the mechanisms underlying H_2_S expression and its regulation of mitochondrial integrity dynamics, as well as the potential role of H_2_S in neuropathological conditions, there is a major gap in understanding how H_2_S levels fluctuate with epileptogenesis. This is a missed opportunity; gasotransmitters are therapeutic targets in epilepsy. For example, the carbonic anhydrase inhibitor, acetazolamide is an approved antiseizure drug, and low levels of CO_2_ have been linked with asynchronous neuronal firing and higher seizure propensity [34]. One of the major barriers to clearly defining the role of H_2_S in the brain has been the prior inability to precisely measure H_2_S concentrations in isolated tissues [35]. While prior reports have indirectly assessed the role of H_2_S on cognitive function in rodents with chemically kindled seizures [36], no study has yet quantified the progressive changes in H_2_S levels in the brain during kindling itself. Furthermore, little work has been conducted to establish how acute seizures influence H_2_S levels in the brain and periphery (i.e., plasma). We thus sought to demonstrate whether H_2_S and downstream enzymatic metabolites were dynamically altered by seizures [37]. The acute maximal electroshock (MES) and 6 Hz 44 mA seizure models were selected to assess changes in H_2_S expression after a single evoked generalized tonic–clonic or focal seizure, respectively. These two models are frontline screening assays of the Epilepsy Therapy Screening Program of the U.S. National Institute of Neurological Disorders and Stroke [38,39]; therefore, studies to precisely quantify acute changes in H_2_S expression in these models is critical to potentially discover new therapies for epilepsy. Kindling effectively models epileptogenesis on a protracted timescale [40], allowing for the evaluation of changes in neuropathology or gene expression with tight control of timing between neurological insults. Therefore, the chronic 60 Hz corneal kindling model was used to assess disease-related changes in H_2_S expression. Lastly, we investigated evidence of dynamic changes in mitochondrial integrity-related protein and gene expression during the early corneal kindling period, which may clarify the role of neurodegeneration and oxidative damage in the onset of spontaneous seizures. 

## 2. Results

### 2.1. Plasma H_2_S Levels Are Reduced Following an Acutely Evoked Maximal Electroshock, But Not in a 6 Hz 44 mA, Seizure

**Figure 1:**
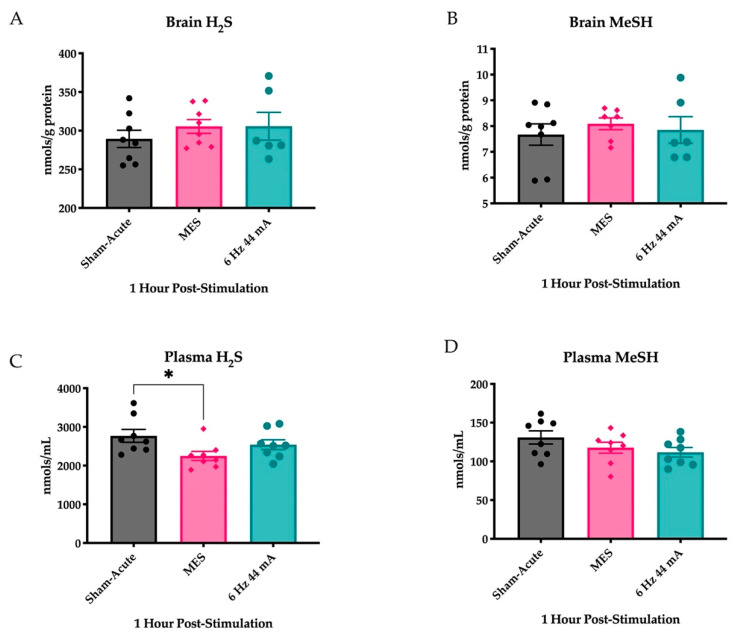
Acutely evoked seizures do not lead to significant changes in H_2_S levels in isolated whole brain tissues but do lead to changes in H_2_S levels in plasma 1 h after electrical stimulation of male CF-1 mice (n = 8 mice/stimulation group). Data are presented as mean +/− S.E.M. (**A**) The levels of H_2_S are not significantly altered in isolated whole mouse brains 1 h after transcorneal stimulation to induce either an MES generalized tonic–clonic seizure or 6 Hz 44 mA focal seizure. (**B**) The levels of MeSH are not significantly altered in isolated whole mouse brains 1 h after transcorneal stimulation to induce either an MES generalized tonic–clonic seizure or 6 Hz 44 mA focal seizure. (**C**) The levels of H_2_S are significantly reduced in plasma collected 1 h after transcorneal stimulation with an MES generalized tonic–clonic seizure. There is no significant reduction in H_2_S levels in the plasma of mice that received a 6 Hz 44 mA focal seizure stimulation. * Indicates significant difference from sham-stimulated mice, *p* < 0.05. (**D**) The levels of MeSH are not significantly altered in the plasma of mice collected 1 h after transcorneal stimulation to induce either an MES generalized tonic–clonic seizure or 6 Hz 44 mA focal seizure.

To address whether a generalized MES or focal 6 Hz 44 mA seizure differentially altered H_2_S and its methylated metabolite methyl sulfide (MeSH) levels, we quantified the levels within the whole brains and plasma from mice collected 1 h after transcorneal stimulation. No significant differences in the levels of H_2_S were apparent from sham between MES and 6 Hz 44 mA stimulated mice (Figure 1A; F_(2,18)_ = 0.6052, *p* > 0.5) or MeSH (Figure 1B; F_(2,18)_ = 0.2973, *p* > 0.7) within the whole brain 1 h after acute transcorneal stimulation. Conversely, plasma levels of H_2_S were significantly reduced following an electrical seizure stimulation (Figure 1C; F_(2,21)_ = 3.518, *p* < 0.05), with post hoc Dunnett’s tests demonstrating a significant reduction in H_2_S levels only in MES-stimulated mice (*p* < 0.05). However, no such significant differences were observed in the plasma levels of MeSH following electrical stimulation (Figure 1D; F_(2,21)_ = 1.808, *p* > 0.1). Follow up studies are needed to determine if the short-term reductions in plasma H_2_S levels, but not MeSH levels, may be a reliable biomarker of an acute generalized tonic–clonic seizure. 

### 2.2. Corneal Kindling Acquisition in Wild-Type Experimental Mice

**Figure 2:**
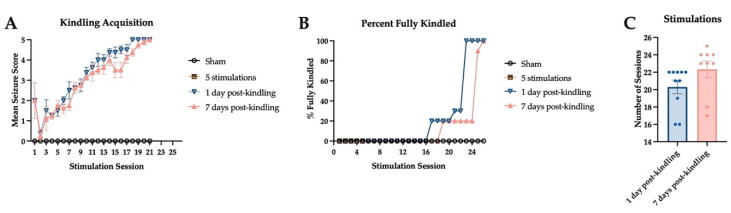
The acquisition of the fully kindled state was no different between the experimental groups used for assessment of H_2_S levels in isolated mouse brains during or after the corneal kindling procedure. Male CF-1 mice were electrically kindled twice daily with a 3.0 mA 60 Hz electrical stimulus delivered bilaterally to anesthetized corneas; sham-kindled mice were similarly handled, but no stimulation was delivered. (**A**) The mean seizure score of mice (+/− S.E.M.) that were euthanized 1 day after attaining the fully kindled state was not different from mice that were euthanized 7 days after reaching the kindling criterion. (**B**) The percent of fully kindled mice in each stimulation cohort was not significantly different. (**C**) There was no significant difference between the two fully kindled cohorts in the numbers of stimulation sessions needed to reach kindling criterion (mean +/− S.E.M.).

Male CF-1 mice were divided into three stimulation groups that underwent the corneal kindling procedure and a sham-kindled cohort. There was no significant difference in the development of the fully kindled state in mice that were euthanized 1 day or 7 days post-kindling (Figure 2); mice that were euthanized after five corneal stimulations (i.e., on the third experimental day) also tracked alongside the two kindling groups until the time of euthanasia. The fifth stimulation was selected as a time point that precedes the onset of secondarily generalized focal seizures [41]. There was no significant difference in the percent of fully kindled mice in both kindling groups; both groups attained 100% of animals fully kindled by 26 stimulations (Figure 2B). Furthermore, the number of stimulations needed to attain fully kindled status was no different between the mice that were euthanized 1 day or 7 days post-kindling (Figure 2C). 

### 2.3. The Corneal Kindling Process Does Not Significantly Impact H_2_S-Related Gene Expression 

**Figure 3:**
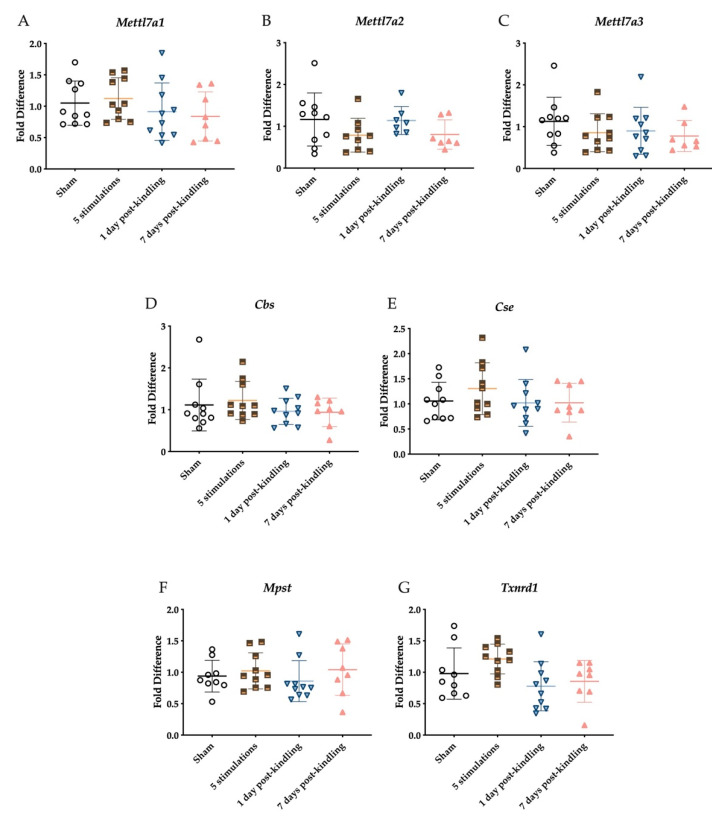
RT-qPCR analysis of genes related to H_2_S production and metabolism in the whole right hemisphere of brains from male mice after corneal kindling. All levels were normalized to the expression of the housekeeping gene, GUSB, and fold difference was normalized to the sham-kindled group. Data are presented as mean +/− S.E.M. (**A**–**F**) No significant changes in expression were observed in genes related to H_2_S metabolism, including the Mettl7a isoforms. There were otherwise no significant changes were observed in CBS, CTH, and MPST upon full acquisition of the kindled state. (**G**) There was a significant main effect of kindling on the expression of TXNRD1 (F_(3,33)_ = 2.917, *p* < 0.05), although post hoc differences between groups were not observed (and thus no * indicated).

H_2_S undergoes methylation via methyl transferase as protein 7B does, and our group has previously identified several mouse orthologs (Mettl7a1, Mettl7a2, and Mettl7a3, Mettl7b, Mettll7b3) to study [42]. At the time of this study, only Mettl7a1, Mettl7a2 and Mettl7a3 primers were commercially available and thus quantifiable. These primary genes associated with H_2_S synthesis and metabolism were quantified during kindling progression (Figure 3). Slight alterations in gene expression of three Mettl7a isoforms were observed across the different treatment groups compared to the sham kindling group (Figure 3A–C); however, the changes were not significant. Similarly, CBS and MPST expression were altered slightly across the different treatment groups but were not significantly different from sham-kindled mice (Figure 3D,E). Finally, the expression of TXNRD1 was significantly impacted by corneal kindling (Figure 3G; F_(3,33)_ = 2.917, *p* < 0.05), but there were no post hoc differences between experimental and sham-kindled mice. 

### 2.4. Repeated Corneal Stimulation Leads to Transient Reductions in Plasma H_2_S Levels

**Figure 4:**
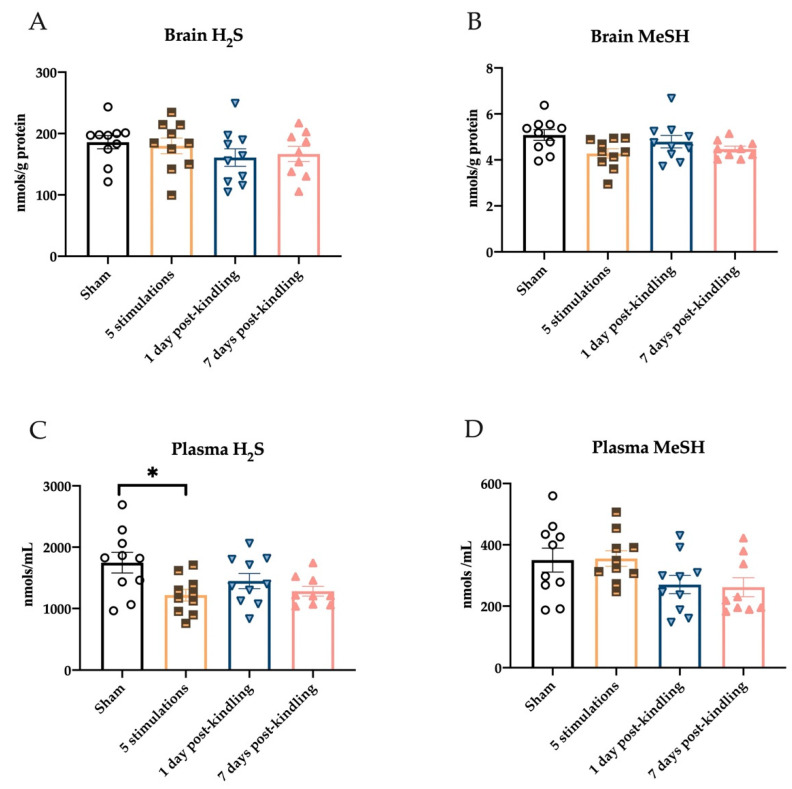
The levels of H_2_S and MeSH are quantified in the brains and plasma of male mice with time after induction of the corneal kindled model of temporal lobe epilepsy. Data are presented as mean +/− S.E.M. (**A**) Changes in whole brain H_2_S levels were not significantly affected by the 60 Hz transcorneal stimulation needed to induce the fully kindled state. (**B**) The levels of MeSH in the whole brain were not significantly reduced post-kindling. (**C**) The levels of H_2_S in purified plasma were significantly reduced in mice euthanized 1 h after the fifth transcorneal stimulation, but levels were no different from sham-kindled mice when assessed 1 day or 7 days post-kindling. (**D**) The levels of MeSH in the plasma were not significantly different from sham-kindled mice. * Indicates significant difference from sham-kindled mice, *p* < 0.05.

To determine whether H_2_S and MeSH levels were only transiently or chronically impacted by corneal kindling, we next quantified the levels in both plasma and whole brain homogenates after five stimulations 1 day post-kindling or 7 days post-kindling. Levels of H_2_S in the brains of mice at these three experimental conditions were not significantly altered with time in the kindling process (Figure 4A; F_(3,35)_ = 0.8471, *p* > 0.4). Brain MeSH levels were also not significantly different between experimental groups and sham-kindled mice, but there was a strong trend for a main effect of stimulation (Figure 4B; F_(3,35)_ = 2.683, *p* = 0.062). However, consistent with the acute seizure induction models studies (Section 2.1), there was a strong and significant main effect of seizure stimulation on plasma H_2_S levels (Figure 4C; F_(3,35)_ = 3.686, *p* < 0.05), with post hoc tests revealing significant reductions in plasma H_2_S levels following the fifth transcorneal stimulation (*p* < 0.01). There was, however, no significant change in plasma MeSH levels in the stimulated cohorts (Figure 4D; F_(3,35)_ = 2.490, *p* = 0.076). Thus, plasma H_2_S levels are consistently and significantly reduced following a seizure acutely evoked by a 60 Hz, but not 6 Hz, corneal stimulation, as both the MES and 60 Hz corneally kindled mouse models exhibited robust reductions in plasma H_2_S levels 1 h after stimulation.

**Figure 5:**
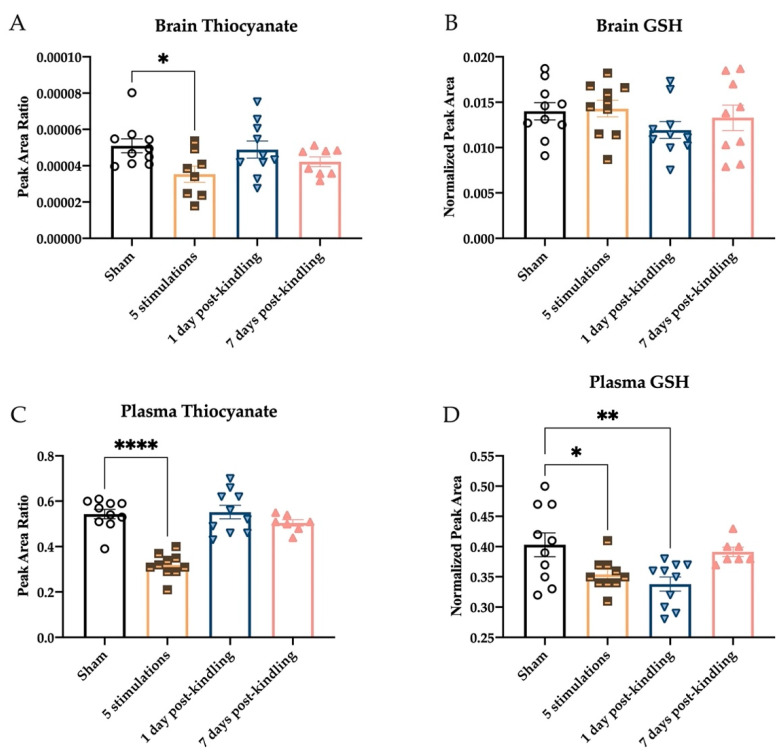
The levels of thiocyanate and reduced glutathione (GSH) were quantified in the brains and plasma of male mice with time following induction of the corneally kindled model of temporal lobe epilepsy. Data are presented as mean +/− S.E.M. (**A**) Changes in whole brain thiocyanate levels were significantly reduced 1 h after the 60 Hz transcorneal stimulation needed to induce the fully kindled state, but there was no difference from sham-kindled mice when assessed 1 day and 7 days post-kindling. (**B**) The levels of GSH in the whole brain were not significantly different from those of sham-kindled mice when assessed after the fifth stimulation or 1 day or 7 days post-kindling. (**C**) The levels of thiocyanate in purified plasma were significantly reduced in mice euthanized 1 h after the fifth transcorneal stimulation, but the levels were no different from those of sham-kindled mice when assessed 1 day or 7 days post-kindling. (**D**) The levels of GSH in the plasma were significantly reduced in mice euthanized 1 h after the fifth transcorneal stimulation, as well as in mice euthanized 1 day after the acquisition of the fully kindled state. However, levels of GSH were no different from those of sham-kindled mice when assessed 7 days post-kindling. * Indicates significant difference from sham-kindled mice, * *p* < 0.05, ** *p* < 0.01, **** *p* < 0.0001.

The levels of thiocyanate and reduced glutathione (GSH) were similarly assessed during the kindling process to determine whether H_2_S metabolism and overall tissue redox status were also affected by kindling (Figure 5). Thiocyanate levels were reduced in the brains of kindled mice (Figure 5A; F_(3,32)_ = 2.90, *p* = 0.050), and post hoc tests revealed that this decrease attained significance after the fifth corneal stimulation (*p* < 0.05), further demonstrating that acute seizure history affects brain H_2_S biosynthesis and metabolism. However, whole brain GSH levels were not significantly impacted by kindling (Figure 5B; F_(3,35)_ = 1.026, *p* > 0.3), suggesting that changes in redox status were not associated with corneal kindling. Whether hippocampal GSH levels would have been affected remains to be further determined. Within plasma, there was a significant effect of seizure history on thiocyanate levels (Figure 5C; F_(3,33)_ = 25.76, *p* < 0.0001); post hoc tests again revealed that the most significant reductions in thiocyanate levels were detected after the fifth stimulation (*p* < 0.0001). Plasma GSH levels were also markedly reduced in mice that had a history of chronic seizures (Figure 5D; F_(3,33)_ = 5.276, *p* < 0.01). Interestingly, levels of GSH in the plasma of mice were reduced following the fifth corneal stimulation (*p* < 0.05), as well as 1 day after attaining the fully kindled state (*p* < 0.01), but there was no difference from sham for fully kindled mice 7 days post-kindling (*p* > 0.8). Thus, brain and plasma thiocyanate levels, as well as plasma GSH concentrations, are transiently reduced with a history of repeated seizures. 

### 2.5. Corneal Kindling Attenuates Inner Mitochondrial Membrane-Associated Gene Expression 

**Figure 6:**
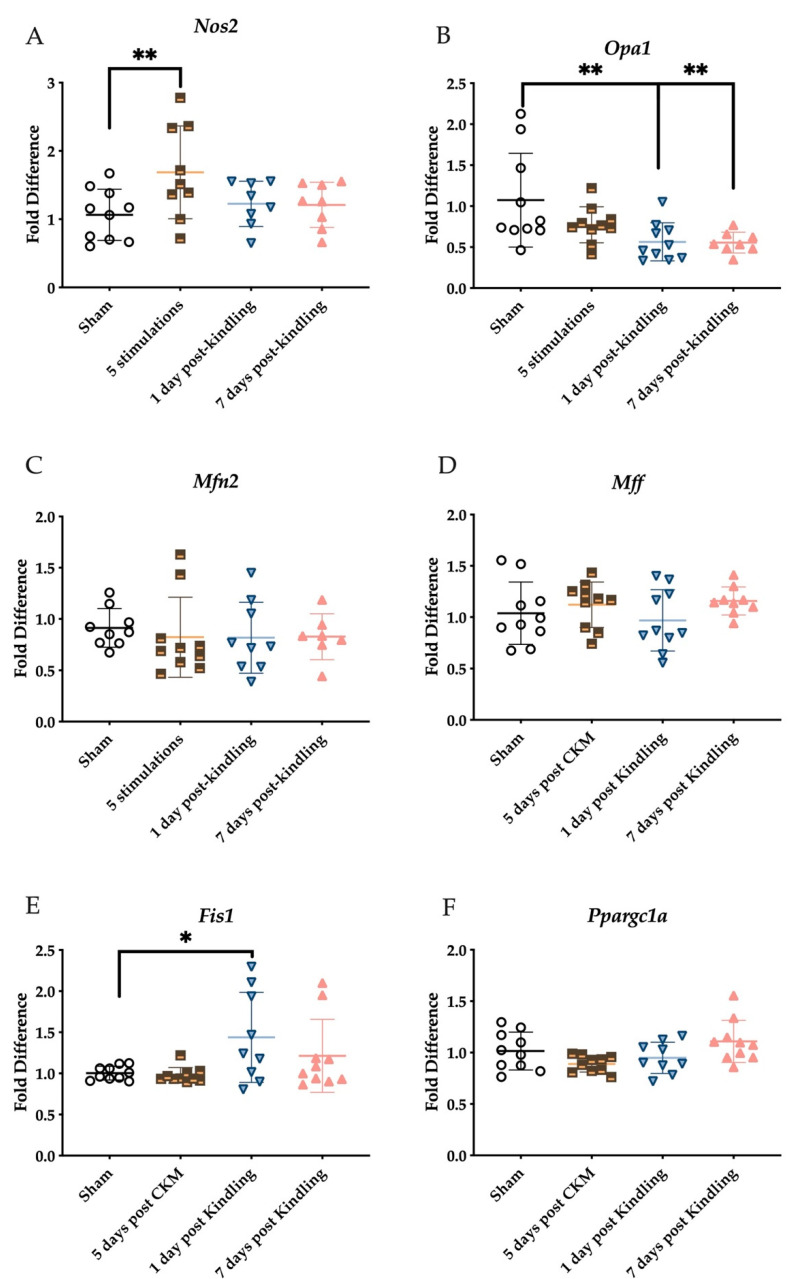
RT-qPCR analysis of genes related to mitochondrial stress normalized to the reference (housekeeping) gene, GUSB, in the whole right hemisphere of the mouse brain following sham or corneal kindling. Data are presented as mean +/− S.E.M. (**A**) The expression of NOS2 was significantly upregulated in the whole right hemisphere of the mouse brain after five stimulations of corneal kindling but not altered after the acquisition of the fully kindled state. (**B**) OPA1 gene expression was significantly downregulated in the whole hemisphere of the mouse brain after full acquisition of the kindled state but minimally altered after five stimulations, while kindling did not significantly alter the expression of (**C**) MFN2 in the whole hemisphere. (**D**) Mff gene expression in the whole hemisphere was not significantly altered by the corneal kindling process. (**E**) However, expression of FIS1 was significantly affected by the corneal kindling (F_(3,35)_ = 3.519, *p* < 0.05), with a post hoc test demonstrating elevated expression at 1 day post corneal kindling (*p* < 0.05) in the whole right hemisphere. (**F**) The expression of PPARGC1A was significantly different with kindling (F_(3,35)_ = 3.329, *p* < 0.05), but there were no post hoc differences between groups and sham-kindled mice at any time point. * Indicates significant difference from sham-kindled mice, *p* < 0.05; ** indicates significant difference from sham-kindled mice, *p* < 0.01.

In addition to assessing the biosynthesis and metabolism of H_2_S and general redox status, we quantified the expression of several genes from whole right brain homogenates of mice in each kindling group. We first assessed the expression of NOS2, which contributes to the generation of reactive nitrogen species and cysteine modification underlying mitochondrial signaling and neuroinflammation. There was a main effect of stimulation on NOS2 expression in the whole right hemisphere (Figure 6A; F_(3,31)_ = 3.195, *p* < 0.05). Post hoc tests demonstrated that the brain expression of NOS2 was significantly elevated in mice after the fifth stimulation versus sham-kindled mice (*p* < 0.05), while NOS2 levels in the brain 1 or 7 days after the acquisition of the fully kindled state were no different from sham (Figure 6B; *p* > 0.8). To further determine whether redox status and H_2_S biosynthesis changes were also associated with changes in mitochondrial integrity dynamics, we quantified the expression of genes critically involved in mitochondrial fusion (OPA1, MFN2; Figure 6B,C), fission (FIS1, Mff; Figure 6D,E), and biogenesis (PPARGC1A; Figure 6F). OPA1 gene expression was significantly decreased by the kindling stimulation (Figure 6B; F_(3,34)_ = 4.850, *p* < 0.01), with post hoc Dunnett’s test demonstrating that these reductions were measured after attaining the fully-kindled state (1 day post-kindling, *p* < 0.01; 7 days post-kindling, *p* = 0.0084). Minor alterations in MFN2 gene expression were observed across the different kindling groups, although the changes were not significant (Figure 6C). There was no time-dependent effect of kindling on Mff gene expression in the whole right hemisphere (Figure 6D; F(3,35) = 1.097, *p* > 0.3); However, we detected a significant increase in the expression of mitochondrial fission 1 (FIS1) in response to corneal kindling (Figure 6E; F_(3,35)_ = 3.519, *p* < 0.05), with a post hoc test demonstrating elevated expression at 1 day post corneal kindling (*p* < 0.05). Lastly, we evaluated the expression of the peroxisome proliferator-activated receptor gamma coactivator 1-α (PPARGC1A) gene (Figure 6F). While there was a significant kindling effect (F_(3,35)_ = 3.329, *p* < 0.05) on this gene, there were no post hoc test differences between experimental groups. Thus, our study demonstrates that the expression of several mitochondria-associated genes is altered as a function of the kindling process. 

### 2.6. Mitochondrial Integrity Dynamics Are Altered by Corneal Kindling 

**Figure 7:**
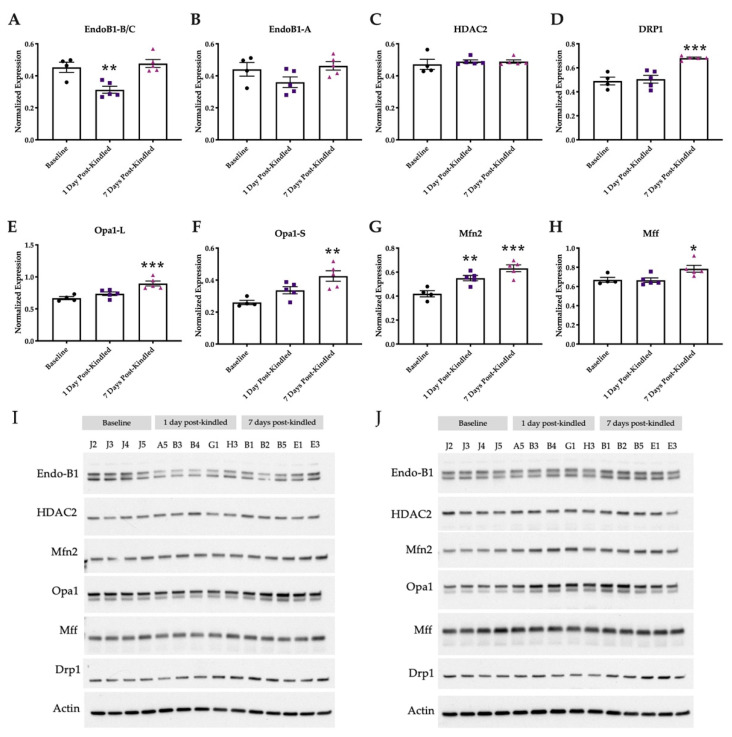
Male mice demonstrate evidence of dynamic changes in mitochondrial membrane integrity-associated protein expression within isolated hippocampus following induction of the corneally kindled model of acquired temporal lobe epilepsy. Left and right hippocampal tissues were micro-dissected at baseline prior to kindling or 1 or 7 days after acquisition of the fully kindled state. (**A**) Expression of the mitochondrial protein Endo-B1-B/C isoform (neuron specific) was significantly reduced 1 day after acquisition of the fully kindled state but returned to baseline levels by 7 days post-kindling. (**B**) Expression of the mitochondrial protein Endo-B1-A isoform (ubiquitous) was not significantly reduced by corneal kindling. (**C**) Expression of HDAC2, which modulates Endo-B1 expression, was not significantly affected by corneal kindling. (**D**) Expression of Drp1, a mitochondrial GTPase, was significantly elevated 7 days after acquisition of the fully kindled state. Drp1 recruitment to mitochondria is necessary and sufficient for mitochondrial fission in periods of cellular stress. (**E**) Expressions of OPA-1 long (OPA1-L) and OPA-1 short (OPA1-S) on the inner mitochondrial membrane with mitofusin 2 (MFN2) on the mitochondrial outer membrane are critical to the maintenance of fusion-competent mitochondria under periods of cellular stress. Expression of the OPA1-L isoform was significantly elevated 7 days after acquisition of the fully kindled state. (**F**) Expression of the OPA1-S isoform was significantly elevated 7 days after acquisition of the fully kindled state. (**G**) Expression of mitofusin (MFN2) was significantly elevated by kindling in general, showing robust increases at both 1 day and 7 days post-kindling acquisition. (**H**) Expression of mitochondrial fission factor (Mff) was significantly elevated 7-days post kindling acquisition. All data are presented as the mean of bands from the left and right hippocampus for all mice in each experimental group, with +/− S.E.M. and all statistical analysis conducted by one-way ANOVA; * indicates *p* < 0.05, ** indicates *p* < 0.01, and *** indicates *p* < 0.001. (**I**) Immunoblot image of micro-dissected right hippocampus. All protein bands of interest were normalized against ꞵ-actin values within the same blot. Animal IDs are listed above each corresponding protein lane. (**J**) Immunoblot image of micro-dissected left hippocampus. All protein bands of interest were normalized against ꞵ-actin values within the same blot. Animal IDs are listed above each corresponding protein lane.

A separate cohort of corneally kindled mice was then used to micro-dissect the whole hippocampus to assess the changes in the expression of mitochondrial membrane integrity dynamics-regulating proteins after establishment of the fully kindled state versus baseline levels. Mitochondrial proteins included Endo-B1 (as isoforms: Endo-B1 B/C—neuron specific; Endo-B1 A—ubiquitous), HDAC2 (which regulates Endo-B1), Drp1, OPA1, MFN2, and Mff (Figure 7). Only Endo-B1-B/C (neuron specific) showed a time-related change in expression (Figure 7A; F_(2,11)_ = 12.47, *p* < 0.01), with post hoc tests demonstrating significant reductions 1 day post-kindling (*p* < 0.01). Neither hippocampal Endo-B1-A (ubiquitous) (Figure 7B; F_(2,11)_= 2.717, *p* > 0.1), nor HDAC2 (Figure 7C; F_(2,11)_ = 0.2972, *p* > 0.7) expression were significantly affected with kindling. The expression of several critical regulators of mitochondrial membrane integrity increased in a time-related manner over baseline, including: Drp1 (Figure 7D; F_(2,11)_ = 17.06, *p* < 0.001), which also showed significant post hoc elevation 7 days-post kindling (*p* < 0.001); OPA1-L (Figure 7E; F_(2,11)_ = 13.36, *p* = 0.0011), which was found to be significantly elevated 7 days post-kindling (*p* < 0.01); and OPA1-S (Figure 7F; F_(2,11)_ = 10.10, *p* < 0.01), which was also significantly elevated 7 days post-kindling (*p* < 0.01). Furthermore, the expressions of both MFN2 and Mff were elevated (F_(2,11)_ = 16.28, *p* < 0.001; F_(2,11)_ = 5.384, *p* < 0.05, respectively). MFN2 levels were significantly elevated at both 1 and 7 days post-kindling (Figure 7G, *p* < 0.001); Mff levels were significantly elevated at 7 days post-kindling (Figure 7H, *p* < 0.05). Thus, corneal kindling is associated with marked time-related changes in the expression of proteins regulating both mitochondrial fission (Drp1 and Mff) and fusion (OPA1 and MFN2), suggesting that mitochondrial stress may be present in the early phases of this model of acquired epilepsy.

## 3. Discussion

This study had two primary objectives. First, we aimed to precisely and directly quantify changes in H_2_S, MeSH, thiocyanate, and GSH in whole brain homogenates and plasma in acute and chronic seizure models evoked in mice. Second, we aimed to define the degree to which mitochondrial membrane integrity-related proteins are dynamically affected with kindling, a model of acquired temporal lobe epilepsy (TLE) [43,44]. We herein demonstrate that acute and repeated seizures lead to transient reductions in plasma H_2_S levels, GSH, and thiocyanate, suggesting that H_2_S may be a novel peripheral biomarker of acute seizures. One major difficulty in prior studies on the role of H_2_S was the inability to precisely measure levels of this gasotransmitter in tissues and plasma samples. In this regard, our present study provides a major advance to further quantitative investigations of the role of H_2_S in preclinical models of disease. Our study is the first to demonstrate rapid reductions in plasma H_2_S levels following an acute MES seizure. The mouse MES test is a well-characterized model of generalized tonic–clonic seizure that is routinely implemented for early drug discovery [37,45]. The MES seizure is substantially differentiated from the pharmacoresistant 6 Hz 44 mA seizure in its engagement of hippocampal and limbic structures [46], as well as a differentiated pharmacological sensitivity [39,46,47,48,49,50]. We herein demonstrate that the generalized MES seizure is also substantially differentiated from the 6 Hz 44 mA focal seizure in the changes of plasma H_2_S 1 h after stimulation. H_2_S may thus be a uniquely sensitive plasma biomarker of severe seizures, and further studies are necessary to confirm this finding. Despite demonstrating the ability to robustly measure H_2_S levels via LC-MS/MS in isolated tissue, H_2_S levels or genes associated with the H_2_S pathway were not dynamically altered in whole brains of corneally kindled versus sham-kindled mice. Conversely, plasma H_2_S levels were significantly decreased after the fifth stimulation but were no different from sham by 7 days post-kindling. As it currently stands, it remains unclear whether H_2_S expression in the hippocampus of mice undergoing corneal kindling is protective of or detrimental to the acquisition of the fully kindled state [41]. 

To further determine the effects of corneal kindling in H_2_S metabolism and overall tissue redox status, thiocyanate and glutathione levels were similarly assessed by LC-MS/MS. Thiocyanate is a detoxification product of cyanide via H_2_S and the transsulfuration pathway catalyzed mainly by 3-MST and rhodanese [51]. After the fifth stimulation, brain and plasma thiocyanate levels were significantly lower compared to sham, further supporting the importance of changes in H_2_S biosynthesis and metabolism with a history of repeated seizures. This trend is consistent after 1 and 7 days of kindling, though the decrease did not remain significant. On the other hand, no significant changes in brain glutathione levels were observed, suggesting that changes in whole brain redox status are perhaps not as important for corneal kindling. 

Importantly, we previously demonstrated that the corneally kindled mouse model is not associated with hippocampal neurodegeneration at the time points currently examined [41], which, together with our present findings, suggests that neurodegeneration is necessary for the onset of oxidative stress and altering H_2_S levels in early epileptogenesis. Taken together, these data suggest that the corneal kindling model is not suitable for monitoring dynamic alterations in H_2_S levels, and neurodegeneration may be a prerequisite. However, this is not to dismiss a role for H_2_S in the onset and progression of epilepsy and mitochondrial membrane integrity dynamics. Nonetheless, augmentation of H_2_S levels may be a novel anticonvulsant strategy. Exogenous application of H_2_S has acute anticonvulsant potential; Efron elegantly described in 1956 a case of a patient whose seizures with olfactory auras could be controlled by exposure to strong and unpleasant odors, including H_2_S [52]. Given that the nasal epithelium is one of the most direct routes of CNS penetrance, it is entirely possible that rapid inhalation of H_2_S by patients to stop acute seizures when paired with wearable seizure prediction devices [53] could be a feasible interventional approach that warrants clinical investigation. 

Healthy mitochondria undergo constant remodeling via fission and fusion to maintain neuronal viability, function, and connectivity. Dysregulation of this process contributes greatly to the pathogenesis of epilepsy and numerous types of nervous system diseases, including in mouse models of TLE [29,54]. Mitochondria are also the predominant source of reactive oxygen species underlying oxidative neuronal damage and neurodegeneration [1], which may further promote epileptogenesis. To better understand the effects of dynamic changes in mitochondria during epileptic seizures, we quantified the relative expression levels of mitochondrial membrane integrity-related proteins by western blot in the isolated hippocampus of the corneally kindled mouse model. We observed time-related increases in the anti-apoptotic protein OPA1 (short and long isoforms), as well as increases in Mff, MFN2, and Drp1 at 7 days post kindling, a time when secondarily generalized seizures are readily evoked in response to the initially benign electrical stimulus. OPA1 short and long isoforms are dynamically regulated by the proteolytic cleavage of eight different mRNAs [55], such that our present discordance between mRNA and protein levels suggests differences in post-transcriptional processing, though localized (i.e., hippocampal) expression of OPA1 (and MFN2) require future probing. Despite a significant increase in Mff protein levels 7 days post-kindling, gene expression was unaltered throughout the kindling process. However, the gene expression of FIS1 was significantly upregulated 1 day post-kindling, though protein modulation was not assessed. Mff and FIS1 are important regulators of mitochondrial fission by independently promoting the recruitment of Drp1 [56,57]. Thus, these findings suggest active engagement of mitochondrial quality control processes during early kindling, but these events may be one contributing mechanism to the absence of neurodegeneration found in this model, despite chronically evoked seizures [41].

Additionally, OPA1 is involved in different critical functions underlying mitochondrial membrane integrity, including maintenance of the respiratory chain and membrane potential [58], cristae organization, and apoptotic signaling [59,60] and is necessary together with MFN2 for membrane fusion to preserve dysfunctional mitochondria [61]. Notably, fusion is prevented in dysfunctional mitochondria by the proteolytic cleavage of OPA1-L to OPA1-S. The OPA1-mediated process of mitochondrial fusion can be rescued with OPA1 overexpression [59], suggesting that the increase in OPA1 presently observed may have been a compensatory response in times of high cellular stress. It should be additionally noted that OPA1 can also signal apoptotic cytochrome c release with the onset of cristae remodeling independent of the process required for the initiation of mitochondrial fusion. Specifically, OPA1 may signal re-fusion of dysfunctional mitochondria, which may preserve neuronal integrity to preserve neuronal tissues. Our present findings shed new insight on the dynamic changes in mitochondrial fission and fusion-associated proteins dynamics in the development of epilepsy. We also herein demonstrate acute reductions in Endo-B-1B/C levels in the early (1 day post-kindling), but not later (7 days post-kindling), time points of corneal kindling. This finding warrants further scrutiny. Interestingly, Endo-B-1B/C knockout mice have greater infarct size and heightened astrogliosis in response to ischemic injury [27], suggesting that the transient changes in Endo-B-1B/C levels presently detected may help to maintain neuronal viability. Because the corneal kindling model is not characterized by neurodegeneration ([41] and Supplemental Figure S1) but is associated with extensive astrogliosis at the time points examined [41], our current studies highlight a potential neuroprotective role for OPA1 and/or Endo-B-1B/C in the context of chronic seizures that is worthy of future study.

Mitochondrial biogenesis is strongly regulated by the PGC1-α (PPARGC1A) signaling cascade. PPARGC1A mRNA levels were not significantly altered post-kindling, but a slight increase in expression after 7 days post-kindling was observed. Nitric oxide (NO) has been reported to modulate mitochondrial biogenesis through cGMP-dependent induction of PGC1-α expression [62]. Higher levels of NO are known to induce mitochondrial fission, potentially resulting in neurotoxicity through morphological disruptions, generation of reactive species, and a decrease in ATP production [63]. Nonetheless, NO and H_2_S have been previously shown to promote neuroprotection following episodes of seizures. We demonstrated an increase in NOS2 expression during kindling, which then returned to baseline upon acquisition of the fully kindled state. The present transient changes in H_2_S levels during the kindling process, coupled with the enhanced levels of NOS2 expression and upregulation of OPA1 isoforms, suggests a high likelihood that anti-apoptotic processes may be recruited to mitigate excitotoxicity in this model of acquired TLE. 

Despite significant advances in our understanding of the role of H_2_S in the pathophysiology of neurological disease, numerous issues remain unresolved. With the application of this newly developed quantitative method to accurately measure levels of H_2_S and other thiol compounds from isolated whole mouse brains in a model of adult-onset acquired TLE, our understanding of the role of H_2_S signaling in the brain, as well as the contributions of mitochondrial integrity dynamics in the pathophysiology of early epilepsy, is now advanced. However, the corneally kindled mouse model, along with other kindling models, is not initially characterized by spontaneous recurrent seizures (SRS) in the early period after kindling acquisition (including the time points examined in this study), suggesting that hippocampal neurodegeneration may be necessary for SRS onset. Further, protein levels of CBS were observed to greatly increase in the hippocampus of mice subjected to kainic acid-induced seizures, another established model of TLE [64]. Future studies should therefore better assess how H_2_S levels are dynamically regulated in discrete brain regions relevant to epilepsy (e.g., through the micro-isolation of the hippocampus) and whether this underlies SRS onset and/or burden.

Given the absence of neurodegeneration in the corneal kindling model versus other mouse models of acquired TLE [41], further work is necessary to precisely interrogate the role of H_2_S and timing of mitochondrial dysfunction that may underlie the development of acquired TLE. Future studies to extend our present findings using a model of adult-onset acquired TLE into syndrome-specific models of epilepsy, such as pediatric genetic epileptic encephalopathies (i.e., SCN1A+/− models of Dravet syndrome) [65,66], could expand the generalizability of our current findings. Our study also highlights the importance of exploring novel drivers of disease to uncover potential new therapeutic targets, such as H_2_S. Notably, this study did not seek to address sex as a biological variable but instead established the feasibility and proof-of-concept quantification of H_2_S levels in rodent models of seizure and epilepsy; therefore, only male mice were used. Through future studies with additional preclinical models of epilepsy, including those that are characterized by SRS or have a genetic component, as well as in female rodents with seizures and epilepsy, the therapeutic potential of targeting H_2_S expression in the brain and regulation of mitochondrial quality control may be better resolved. At that point, such studies will provide improved insight to address whether H_2_S may represent a relevant molecular target for epilepsy. 

## 4. Materials and Methods

**Reagents:** Water, acetonitrile, and acetic acid were all optima grade from Fisher Scientific (Hampton, NH, USA). Monobromobimane, MBB, was from Toronto Research Chemicals (North York, ON, Canada). Trizma HCl, Trizma Base, diethylenetriaminepentaacetic acid (DETAPAC), and ACS reagent grade 5-sulfosalicylic acid dihydrate were from Millipore-Sigma (Burlingon, MA, USA). Tissue was homogenized with a Precellys-24, cooled with a Cryolys cooling unit from Bertin (Rockville, MD, USA). Nunc 400 µL 96-well plates and cap mats were purchased from ThermoFisher (Waltham, MA, USA).

**Animals:** All animal experimentation was approved by the University of Washington Institutional Animal Care and Use Committee under approval number 4387-01 (MBH; approval dates 5 August 2016 and renewal 5 May 2019), University of Washington Public Health Service (PHS) Assurance issued by the Office of Laboratory Animal Welfare (OLAW) assurance number D16-00292 and University of Washington AAALAC accreditation number #000523. Male CF-1 mice (4–8 weeks; Envigo Laboratories, Indianapolis, IN, USA) were housed five mice/cage in a temperature-controlled vivarium on a 14:10 light/dark cycle. Mice had free access to irradiated chow (Picolab 5053) and water, except during periods of behavioral seizure testing. Mice were allowed a minimum of 4 days habituation to the housing facility, given a minimum of 1 h to acclimate to the procedure room prior to all experimentation, and euthanized by decapitation at the completion of all in-life studies. 

**Maximal Electroshock and 6 Hz 44 mA Acute Seizure Models: **The in-life experimental design is illustrated in Supplemental Figure S1. Maximal electroshock seizures (MES), a model of generalized tonic–clonic seizure, were evoked with an acute, 60 Hz 0.2 s 50 mA bilateral stimulation [39]. The 6 Hz 44 mA seizure, a model of focal seizures, was evoked with an acute, 6 Hz 3 s 44 mA bilateral stimulation delivered to anesthetized corneas [50]. Sham-stimulated mice were similarly handled, but no electrical current was delivered. There were n = 8 mice/stimulation or sham used (total n = 24) for acute studies. Whole brain and plasma were collected from mice 1 h after transcorneal stimulation. The whole brain was rapidly removed and flash-frozen on dry ice. Whole brain samples were stored at −80 °C until analytical processing for H_2_S and RT-qPCR. Trunk blood for H_2_S analysis was collected with Li-Heparin as the anticoagulant. Plasma was isolated by 10 min centrifugation at 3000× *g* at 4 °C and stored at −80 °C until analytical processing.

**Corneally Kindled Mouse (CKM) Chronic Seizure Model:** For the 60 Hz corneal kindling protocol, mice (n = 30) were stimulated with an initially benign electrical current (60 Hz, sinusoidal pulse; 3.0 mA) delivered for 3 s via corneal electrodes [18,19,20] or sham-kindled (n = 10). Seizures were scored on a 5-point rating scale consistent with the Racine scale in amygdala-kindled rats and routinely used by our group for corneally kindled mice [18,19,20,21,22,23], wherein 1 = jaw chomping and vibrissae twitching, 2 = head bobbing and Straub tail, 3 = unilateral forelimb clonus, 4 = bilateral forelimb clonus and hind-limb rearing, and 5 = bilateral forelimb clonus and rearing followed by loss of righting reflex. Twice daily stimulations continued for each mouse until it achieved the criterion of five consecutive Racine stage 5 seizures, whereby the mouse was considered “fully kindled.” Any mouse not achieving the fully kindled state was not included for further study. Upon euthanasia at the designated time point after corneal kindling (or sham kindling), the whole brain was rapidly removed and flash-frozen on dry ice. Whole brain samples were stored at −80 °C until analytical processing for H_2_S and RT-qPCR. Trunk blood for H_2_S analysis was collected with Li-Heparin as the anticoagulant. Plasma was isolated by 10 min centrifugation at 3000× *g* at 4 °C and stored at −80 °C until analytical processing.

A secondary cohort of mice (n = 14) was used in the corneal kindling procedure for analysis of mitochondrial protein expression within isolated hippocampus. Hippocampal (left and right) sections were rapidly micro-dissected from the brains of mice at baseline (unstimulated for 3 days after study start) or 1 or 7 days after acquisition of the fully kindled state. Upon euthanasia at the designated time point after corneal kindling (or sham kindling), the brain was rapidly removed and the hippocampus micro-isolated and flash-frozen on dry ice. Hippocampal tissues were stored until homogenization and processing for immunoblot. 

**Immunoblot:** Protein extracts for western blot analysis of isolated the left and right hippocampus were prepared as previously reported [27]. Protein extracts from the left and right hippocampus were separated by SDS-PAGE and transferred to a polyvinylidene fluoride membrane (Immun-Blot; Bio-Rad Laboratories, Hercules, CA, USA). After blocking in phosphate-buffered saline containing 0.1% Tween 20 and 2% casein, the membrane was incubated with primary antibodies in the same buffer. Primary antibodies used were mouse monoclonal Endophilin (Novus, Littleton, CO, USA; NBP2-24733; 1:500), rabbit polyclonal Mff (Proteintech, Rosemont, IL, USA; 17090-1-AP, 1:2000), MFN2 (Abcam, Cambridge, United Kingdom; ab124773, 1:2000), HDAC2 (Sigma-Aldrich, St. Louis, MO, USA; H2663, 1:5000), OPA-1 (clone 18/OPA1, 1: 5000; BD Biosciences, Franklin Lakes, NJ, USA), mouse monoclonal Drp1 (clone 8/DLP1, 1:1000; BD Biosciences, Franklin Lakes, NJ, USA), and mouse monoclonal ꞵ-actin (clone AC-15, 1:10,000; Sigma-Aldrich, St. Louis, MO, USA), as previously reported [29]. Horseradish peroxidase-conjugated, species-specific secondary antibodies (1:2000) were from GE Healthcare (Chicago, IL, USA), and blots were developed with SuperSignal West Pico (ThermoFisher Scientific; Waltham, MA, USA). For quantification, images were digitally scanned, and band intensity from left and right hemisphere blots were measured using NIH ImageJ software [67] and normalized against ꞵ-actin values.

**Limited Immunohistochemistry for NeuN:** The whole brain of a fully kindled (7 days post-kindling) and sham-kindled mouse were processed for qualitative assessment of hippocampal neuron integrity at the conclusion of the corneal kindling protocol (n = 2 mice). Our prior published [41] and unpublished studies demonstrate that corneal kindling is not associated with neurodegeneration at the time points presently examined. Therefore, we performed a limited confirmatory assessment of neuronal density via immunohistochemistry for the neuron-specific marker, NeuN, according to our previously published methods [41,68] to again illustrate that fully kindled mice do not exhibit neurodegeneration at 7 days post-kindling acquisition versus sham-kindled mice (Supplemental Figure S2). 

**Total RNA Isolation and RT-qPCR Analysis of Whole Brain Tissues: **The whole right hemisphere of each brain sample collected from CKM was homogenized in a TRI-reagent using the Precellys bead beater (6800 rpm, 3 × 30 s cycles, 60 s delay), followed by extraction using the RNeasy Lipid Tissue Mini Kit (Qiagen, Germantown, MD, USA) to isolate total RNA. RNA purity (A260/280) was assessed, and yield was determined using the Tecan Spark multimode microplate reader (Mannedorf, Switzerland). Total RNA was synthesized to cDNA by reverse transcriptase using the High-Capacity RNA-to-cDNA Kit (Thermo Fisher Scientific, Waltham, MA, USA). RT-qPCR was then conducted utilizing the Taqman Gene Expression Assays (FAM dye; Thermo Fisher Scientific, Waltham, MA, USA), and gene expression was normalized to the housekeeping gene, GUSB, following the comparative CT method. 

**Synthesis of Standards for LC-MS/MS: **The internal standard, propanethiol bimane, was synthesized by mixing 30 mg of 1-propanethiol with 30 mg of monobromobimane (MBB) in 100 mM pH 9.0 Tris buffer for 1 h in a 37 °C water bath. The product was extracted using three volumes of ethyl acetate that were pooled and dried under nitrogen gas. The product was then re-dissolved in acetonitrile and purified by column chromatography. The silica gel column was loaded with product and washed with water to remove impurities, then eluted with acetonitrile. This product was dried and re-dissolved in acetonitrile, and its identity was verified by high resolution mass spectrometry using an Agilent 6500 Q-TOF. Derivatized hydrogen sulfide and methanethiol standards were prepared similarly.

**Sample Preparation for LC-MS/MS:** All tissue and plasma samples were stored at −80 °C until use. During the derivatization process, samples were handled on ice under light limited to red LED bulbs. All solvents had oxygen reduced by vacuuming while stirring, followed by sparging with nitrogen gas. This solvent degassing process was performed twice. Samples were handled in a Cleantech glove box (2100-2-A) under nitrogen atmosphere with >1%oxygen present until after the derivatization with MBB was complete.

Heart and whole brain tissues transferred to Precellys 2 mL reinforced tubes containing 2.8 mm Qiagen ceramic beads and 1 mL of pH 9.0 tris H_2_S trapping buffer with 0.25 mM DETAPAC. They were homogenized in Precellys-24 cooled by a Cryolys for 3 cycles, 30 s each, at 6500 RPM. The homogenate was then centrifuged at 10,000× *g*, 4 °C, for 10 min. In a 96-well plate, 30 µL of the tissue supernatant or plasma was combined with 30 µL of trapping buffer, along with 10 µL of 10 µM propanethiol bimane internal standard. Then, 25 µL of a 20 mM MBB acetonitrile solution were added. The 96-well plate was then sealed with a cap mat, and derivatization took place in a 37 °C water bath for 30 min. The reaction was quenched by adding 75 µL of 100 mM 5-sulfosalicylic acid. The samples were centrifuged at 4000× *g*, 4 °C for 15 min, and then the supernatant was transferred to a fresh 96-well plate and analyzed by LC-MS/MS.

**LC-MS/MS: **H_2_S, MeSH, thiocyanate and GSH were measured using a Waters Xevo TQ-s ESI mass spectrometer operating in positive ion mode coupled to a Waters Acquity I-class UPLC. The chromatography column used was a Waters (Milford, MA, USA) Acquity UPLC BEH shield C18 column (1.7 µm, 2.1 × 150 mm). There was a 10 µL injection volume, 0.3 mL/minute flow rate, with the column heated to 50 °C. The mobile phases were (A) water with 0.2% acetic acid and (B) acetonitrile with 0.2% acetic acid. The LC method was 8 min long, starting with 99% A and ramped down to 60% A at 1.5 min and 30% at 4 min and back to 99% A at 6 min. The quadrupole MS was run in SRM positive ion mode with the highest intensity mass transitions chosen for each compound. The capillary voltage was set to 2 kV, and cone voltage was set to 40 V, with a source offset of 60 V and a desolvation temperature of 350 °C.

**Calibration Curve: **Standards for H_2_S and MeSH were prepared as stated above. A 5-point calibration curve and three quality control standards were added to every plate and were spiked into 30 µL of a homogeneous plasma pool. Calibration curves for both H_2_S and MeSH produced r^2^ values greater than 0.99 and fell within the accepted limits of the FDA bioanalytical method validation [24].

**Statistics: **Statistical differences in gene expression, protein, and H_2_S levels were assessed by one-way ANOVA, with Dunnett’s post hoc tests. Kindling acquisition rates between experimental cohorts were compared by Friedman test, a non-parametric test of variance with repeated measures, with Dunn’s multiple comparisons post hoc tests. The percentages of fully kindled mice were compared by repeated measures ANOVA, with Dunnett’s post hoc tests. The total numbers of stimulations needed to attain the fully kindled state between the two fully kindled cohorts were compared by t-test. For all statistical measures, *p* < 0.05 was considered significant, and all analyses were performed with Prism version 8.0 or later (GraphPad, San Diego, CA, USA). 

## Data Availability

Data are contained within the article. For additional requests please contact the corresponding author.

## Supplementary Materials

The following are available online at https://www.mdpi.com/article/10.3390/ijms23031434/s1.
