# Supplementary material for: Reductions in Hydrogen Sulfide and Changes in Mitochondrial Quality Control Proteins Are Evident in the Early Phases of the Corneally Kindled Mouse Model of Epilepsy
Source: Int J Mol Sci. 2022 Jan 27;23(3):1434. doi: 10.3390/ijms23031434 (PMC8835945; doi:10.3390/ijms23031434)
Supplement: Supplementary file 1 [file ijms-23-01434-s001.zip › ijms-1556305-supplementary.pdf]

Supplemental Files.

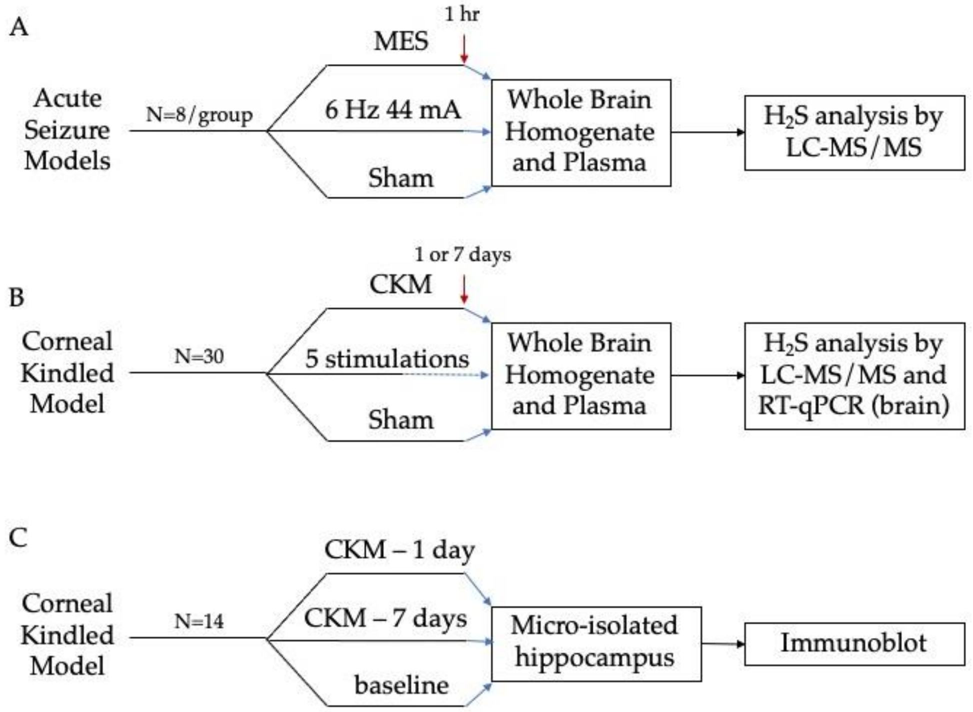

Supplemental Figure S1. Experimental testing procedure for in-life studies in the A) acute seizure models (maximal electroshock(MES) and 6 Hz 44 mA tests) and B–C) corneally kindled mouse (CKM) model of temporal lobe epilepsy.

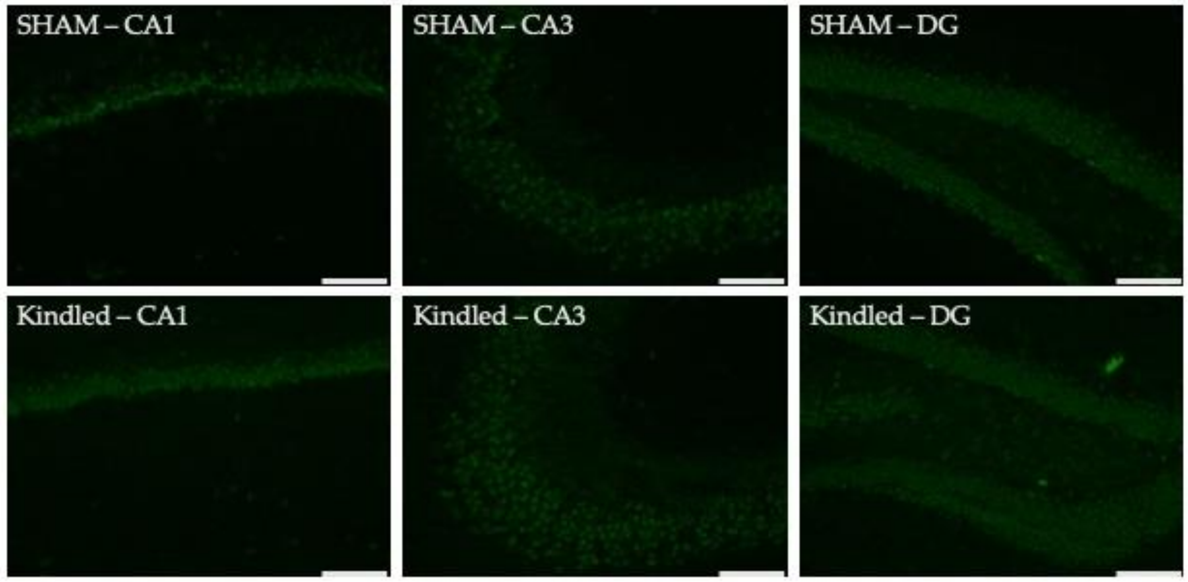

Supplemental Figure S2. A representative photomicroph from the dorsal hippocampus (area CA1, CA3, or dentate gyrus(DG)) of a sham-kindled (top panels) or corneally kindeld male mouse collected 7 days after acqusition of the fully kindled state demonstrates that corneal kindling is not associated with neurodegeneration at the longest time point after kindling is evaluated. The brains of representative mice were rapidly flash frozen in 2-methylbutane and processed for NeuN (green, a marker of neurons) immunoreactivity according to our published protocols [41,68]. Scale bar represents 100 μm.
